# Functional Characterization of the Tau Class Glutathione-*S*-Transferases Gene (*SbGSTU*) Promoter of *Salicornia brachiata* under Salinity and Osmotic Stress

**DOI:** 10.1371/journal.pone.0148494

**Published:** 2016-02-17

**Authors:** Vivekanand Tiwari, Manish Kumar Patel, Amit Kumar Chaturvedi, Avinash Mishra, Bhavanath Jha

**Affiliations:** Division of Marine Biotechnology and Ecology, CSIR-Central Salt and Marine Chemicals Research Institute, G. B. Marg, Bhavnagar (Gujarat), India; Hainan University, CHINA

## Abstract

Reactive oxygen or nitrogen species are generated in the plant cell during the extreme stress condition, which produces toxic compounds after reacting with the organic molecules. The glutathione-S-transferase (GST) enzymes play a significant role to detoxify these toxins and help in excretion or sequestration of them. In the present study, we have cloned 1023 bp long promoter region of tau class GST from an extreme halophyte *Salicornia brachiata* and functionally characterized using the transgenic approach in tobacco. Computational analysis revealed the presence of abiotic stress responsive cis-elements like ABRE, MYB, MYC, GATA, GT1 etc., phytohormones, pathogen and wound responsive motifs. Three 5’-deletion constructs of 730 (GP2), 509 (GP3) and 348 bp (GP4) were made from 1023 (GP1) promoter fragment and used for tobacco transformation. The single event transgenic plants showed notable GUS reporter protein expression in the leaf tissues of control as well as treated plants. The expression level of the GUS gradually decreases from GP1 to GP4 in leaf tissues, whereas the highest level of expression was detected with the GP2 construct in root and stem under control condition. The GUS expression was found higher in leaves and stems of salinity or osmotic stress treated transgenic plants than that of the control plants, but, lower in roots. An efficient expression level of GUS in transgenic plants suggests that this promoter can be used for both constitutive as well as stress inducible expression of gene(s). And this property, make it as a potential candidate to be used as an alternative promoter for crop genetic engineering.

## Introduction

Abiotic stresses are deteriorating for crop productivity as well as quality and are major challenge for sustainable agriculture. Almost all stresses lead to the generation of secondary stressors within the plants, the reactive oxygen species (ROS) which cause oxidative damages to cellular macromolecules and organelles structures. The common examples of these ROS are superoxide radical (O^∙-^_2_), singlet oxygen (^1^O_2_), hydrogen peroxide (H_2_O_2_) and hydroxyl radical (OH^∙^) [1]. These ROS molecules act as a homeostatic signal initiator in plants when accumulated at the lower concentration, but are lethal to the cells if crossed above the homeostatic level. Plants evolved with the different homeostatic mechanisms to maintain ROS level below the alarming concentration exemplified with several enzymatic and non-enzymatic antioxidative pathways [2]. The primary antioxidative pathways involve nonenzymatic antioxidants (ascorbate, glutathione, carotenoids, tocopherols etc.) and detoxifying enzymes (superoxide dismutase, SOD; catalase, CAT; peroxidases etc.). The oxidation-reduction state of members of the ascorbate-glutathione cycle or some specific enzymes such as glutathione-*S*-transferases involve in the maintenance of cellular redox state and finally in stress acclimations [3].

Glutathione-*S*-transferases (GSTs) are ubiquitous enzymes found in entire biological kingdom either prokaryote or eukaryote. GSTs catalyze nucleophilic fusion of reduced glutathione (GSH; a tripeptide Glu-Cys-Gly) with electrophilic and hydrophobic toxic molecules, generated under stress, to convert them in non-toxic and soluble conjugates [4–5]. These soluble conjugates are then either excreted or compartmentalized in the vacuoles mediated by ATP binding cassette (ABC) transporters for their degradation [4]. All GSTs have two domains one on each ends, a conserved N-terminal domain named G-site for binding of GSH and comparatively less conserved C-terminal domain, the H-site for interaction of the hydrophobic toxic molecules [4]. Other functions of GSTs were reported as peroxidases, isomerases and thiol-transferases mediated by reduced GSH and non-enzymatic functions like signaling modulation [5]. A functional GST enzyme is a dimer either homodimer or heterodimer encoded by different genes of the same class [5]. GSTs play an important role in various metabolic pathways, involved in detoxification of oxidative lipid peroxide metabolites, hormone metabolism and functioning of dehydroascorbate [6–7]. They act as a carrier protein in anthocyanin vacuolar transport, signaling component of UV-light stress and phytohormone signaling during plant growth and development [8–10].

In plants, this superfamily of the enzyme is encoded by a large number of genes. The Arabidopsis genome constitutes 55 genes of GST superfamily [11] while that of in rice 52 genes [12]. Plant GSTs are categorized into eight classes named as tau, phi, theta, zeta, lambda, DHAR, tetrachloro-hydroquinone dehalogenase and microsomal GSTs based on sequence similarity, intron number & position in the genome, and immunoreactivity [4, 11]. The phi and tau classes of GSTs are exclusively plant specific and stress inducible, playing their role in xenobiotic-detoxification and defense-related secondary metabolism [5, 11]. Expression of GSTs has been reported to be differentially regulated by a number of abiotic stresses, phytohormones, pathogens and fungal elicitors [12]. In recent years, evident reports showed that over-expression of tau class *GSTs* (*GSTUs*) increased the herbicide and salinity tolerance of transgenic plants [13–14]. Numerous studies on GSTUs indicated that it plays a protective role against abiotic stresses, including detoxification reactions and antioxidative glutathione peroxidase-like functions [15].

Most of these studies are limited to the GSTUs from the glycophytic plants. Halophytes evolved with unique adaptation to survive and complete their life cycle under extreme saline soil and physiological osmotic stress conditions. Dicotyledonous halophytes growing under extreme habitat showed a similar rate of biomass synthesis as the glycophytes under optimal growing conditions [16]. This is the distinctive plasticity in halophytes which maintain their normal growth rates despite of high energy demand, attributed to their unique ability of efficient sequestration of Na^+^ and Cl^-^ ions and its utilization as an osmoticum. Halophytic proteins have a higher percentage of acidic and hydrophilic amino acid residues than that of their glycophytic homologs [17]. Comparative transcriptome analysis of *Arabidopsis thaliana* and *Thellungiella halophila* using microarrays and expressed sequence tags (ESTs) exhibited that many stress-related genes constitutively expressed at higher level in *T*. *halophila* than that of their homologs in *A*. *thaliana*. This finding suggests that halophytes may have a more efficient transcriptional regulatory network for stress acclimation and maintain higher transcript level of stress responsive genes [18–19]. We believe that this higher level of stress responsive transcripts accumulation in halophytes is a strategy to avoid unnecessary energy consumption in switching-on/-off of transcription with fluctuating environmental conditions. One of the best examples of these halophytes is *Salicornia brachiata*, which is a leafless, succulent extreme eudicot halophyte, growing in salt marshes. It contains unique oligosaccharides, metabolites, S-rich amino acids and, requires salt (NaCl) for *in-vitro* growth and has the ability to accumulate salt up to 40% of its dry weight [20–24]. Total of 930 expressed sequence tags (ESTs) were identified through suppressive subtractive hybridization from *S*. *brachiata* which were differentially expressed in salinity stress [25]. Out of these, 90 genes were unknown and hypothetical [25] and some of them are recently studied for their role in stress tolerance in bacteria as well as in plants [17, 26–30]. Additionally, a number of known genes and their promoters from *S*. *brachiata* when introduced in heterologous plants, have proved that they may serve as a potential candidate stress responsive genes or promoters for crop genetic engineering [31–34].

Genetic engineering of crops using transgenic approach is a rapid method of crop improvements. In the last two decades, a number of genetically engineered plants were raised and showed enhanced protection from the stresses [35–36]. However, some of the genetically engineered plants come up with undesirable side effects too, like low yield [37], delayed growth [38] and dwarfism [39] and none the least, the ethical issues by some agencies. These phenotypes are the result of energy consuming high level transgene expression driven by constitutive viral promoters and ectopic expression of the transgenes [40]. Alternative to constitutive promoters, stress-inducible promoters from different plants were cloned and characterized for their efficiencies to drive the transcript expression [34]. Use of these promoters to drive stress responsive transgene expression in host plants will reduce the risk of phenotypic abnormalities as it drives the expression only under the stress conditions while under normal conditions the transcript expression level are either very low or negligible. This may endure better stress tolerance in the transgenic plants.

Identification, cloning and characterization of putative promoters from the halophytes will provide an alternative of stress-inducible promoters to be used to drive expression of potential transgenes in genetically engineered crops for better tolerance under multiple abiotic stresses. Plant tau-class GSTs express especially in response to different abiotic stress conditions suggesting a common transcriptional regulation under different abiotic stresses. Therefore, in the present study, the putative promoter region (1023 bp upstream from ATG) of *SbGSTU* was cloned and analyzed *in-silico* for identification of *cis*-regulatory motifs. The promoter was serially deleted from 5’-end and fragments were cloned into pCAMBIA1301 plant expression vector by replacing the CaMV35S promoter upstream to the GUS reporter gene. Transgenic tobacco plants in T_1_ generation having the single copy of transgene integration were used for quantification of GUS expression under normal or salinity and osmotic stress conditions for determination of the optimal length of *SbGSTU* promoter to drive efficient reporter gene expression.

## Materials and Methods

### Plant materials and stress treatments

The *S*. *brachiata* Roxb. seeds were germinated in garden soil under controlled laboratory conditions. One-month-old seedlings were harvested, frozen into liquid nitrogen and stored at -80°C for further uses. Tobacco (*Nicotiana tabacum* cv. Petit Havana) seeds were germinated on MS (Murashige and Skoog) basal medium supplemented with 3% sucrose, pH 5.8, and solidified with 0.8% agar in a petri-dishes of 90 mm diameter for three weeks and then transferred to the same media in culture bottle for further growth. Leaf tissues of 4-weeks old plants were used for the genetic transformation.

The seeds of the transgenic tobacco lines harboring single copy insertion of the transgene in T_1_ generation were germinated on the above said MS basal media supplemented with 20 mg l^-1^ hygromycin. Three week old seedlings were then transferred to the hydroponic medium (0.5X Hoagland solution) and allowed to grow for 2 weeks and the hydroponic medium was replaced with fresh medium at each 4^th^ day. Thereafter, for salinity stress, plants were transferred to Hoagland solution (0.5X), containing 200 mM NaCl for 6 h and 12 h. For osmotic stress treatment, plants were transferred to 10% (w/v) PEG-6000 in 0.5X Hoagland solution for a period of 3- and 6-days. The time point of the stress treatment was determined based on the visible wilting in plants. Plants growing on the 0.5X Hoagland solution without any stress treatment for the same period as indicated above were chosen as control. Leaf, shoot and root tissues of transgenic tobacco were harvested separately, immediately frozen in liquid nitrogen and stored at -80°C for further experiments.

### Isolation of 5’-upstream *cis*-acting elements and *in silico* analysis

Genomic DNA was extracted from *S*. *brachiata* shoots and tobacco seedlings using a modified cetyltrimethylammonium bromide (CTAB) method. The genomic DNA concentration was determined with a NanoDrop ND-1000 spectrophotometer and the quality was checked by 0.6% agarose gel electrophoresis. For isolation of the 5’-upstream promoter region of the *SbGSTU*, primers GSTP F and GSTP R (S1 Table) were designed from the *SbGSTU* sequence (accession no.: EU295484). The putative promoter region of 1023 bp (-1028 to -6 bp) upstream to the ATG was amplified from the genomic DNA of *S*. *brachiata* using standard PCR conditions (S1 Table) and sequenced. *In silico* analysis and homology searches for cis-regulatory elements in the promoter were performed using the online programs PLACE [41] and PlantCARE [42] and categorized into different classes based on their sequence similarity.

### Construction of promoter expression vector and tobacco transformation

The expression efficiency of the *SbGSTU* gene promoter (*SbGSTU*-P) under stress was functionally validated by a transgenic approach. Vector constructs for plant transformation were prepared by replacing the CaMV35S promoter upstream of the GUS gene with the putative *SbGSTU*-P in pCAMBIA1301 using cloning strategy as described by Tiwari et al. [34]. The putative *SbGSTU*-P region (1023 bp; -1028 to -6 bp upstream to ATG) was amplified using a primer set GP1 F–GP R containing restriction sites *Sal*I and *Bgl*II (S1 Table), cloned into the pGEM-T Easy vector (*SbGSTU-P–pGEM*) and confirmed by sequencing. The *SbGSTU-P–pGEM* vector was digested with *Sal*I and *Bgl*II, and sub-cloned into pCAMBIA1301, and the resultant construct was named GP1. Similarly, three more 5’-deletion constructs of *SbGSTU*-P along with the 5’-UTR were prepared and named GP2 (730 bp; -735 to -6 bp upstream to ATG), GP3 (509 bp; -514 to -6 bp upstream to ATG) and GP5 (348 bp; -353 to -6 bp upstream to ATG), respectively, using different primer sets (S1 Table). All promoter constructs were mobilized into *Agrobacterium tumefaciens* LBA4404 and transformed into tobacco leaf discs using a standard protocol [43]. Primary transformants (T_0_) were raised, transferred to soil and allowed to grow in a greenhouse. Seeds were harvested and used for analysis in the next generation.

### Confirmation of transgene integration and determination of copy number

Seeds from five independent transgenic lines of each transformation were randomly selected and germinated on MS basal medium supplemented with 20 mg l^-1^ hygromycin. Genomic DNA was isolated from the seedlings and transgene integration was confirmed by PCR amplification of the *hptII* gene using gene-specific primers (S1 Table). Transgene copy number was determined by Southern hybridization; for this, 15 μg of genomic DNA from each transgenic line were digested with *Bam*HI overnight. Digested DNA fragments were separated on 0.7% agarose and blotted onto a Hybond (N^+^) membrane (Amersham Pharmacia, UK) using alkaline transfer buffer (0.4 N NaOH with 1 M NaCl). A digoxigenin (DIG)-11-dUTP-labeled *GUS* gene specific DNA probe was synthesized by PCR according to the manufacturer’s protocol (Roche, Switzerland) using primer set GQF–GQR (S1 Table). Hybridization was carried out at 42°C overnight in DIG EasyHyb buffer solution (Roche, Switzerland). The hybridized membrane was detected using CDP-Star as substrate (Roche, Switzerland) and signals were visualized on X-ray film after 30 min. exposure. Lines showing single copy transgene integration were used further for the analysis.

### GUS histochemical and quantitative assay

The GUS histochemical assays of 5-week-old seedlings of single copy transgenic tobacco lines were performed using the *β*-Glucuronidase Reporter Gene Staining Kit (Sigma-Aldrich, USA). Plant seedlings were freshly taken from the hydroponic medium, washed with water, blotted and incubated in the staining buffer overnight at 37°C in the dark. Quantitative analyses of GUS activity in leaf, stem and root tissues were carried out according to Jefferson et al. [44]. For quantitative assay, tissues from control and treated plants were homogenized in 500 μl of GUS extraction buffer (50 mM NaPO4 buffer, pH 7.0, 10 mM EDTA pH 8.0, 0.1% sodium lauryl sarcosine, 0.1% Triton X-100 and 10 mM 2-mercaptoethanol) and centrifuged at 15,700 g for 15 min at 4°C. The supernatant was transferred to a fresh tube, and GUS activity was assayed with 1 mM (4-methylumbelliferyl-*β*-D-galactopyranoside (MUG) solution in GUS extraction buffer. Crude protein extract (50 μl) was added to 450 μl of MUG assay buffer and immediately 100 μl aliquots were taken and added to 900 μl of stop buffer (0.2 M Na_2_CO_3_) for a reagent blank. Before start of the experiment, it was taken care that the final concentration of the MUG in MUG assay buffer should be 1 mM. The reaction mixture was incubated at 37°C for 1 h, and thereafter 100 μl of the reaction mixture was added to 900 μl of stop buffer. The fluorescence level of the breakdown product, 4-methylumbelliferone (4-MU), was detected in an LB-970 Fluorescence Reader Twinkle (Berthold Technologies, Germany) using excitation/emission filter of 355/460 nm for 4-MU. The concentration of 4-MU was determined by a standard curve. The total protein concentration in crude sample extracts was determined using the Quick StartTM Bradford Protein Assay Kit (Bio-Rad, USA). GUS activity was calculated as pmol of 4-MU min^-1^ mg^-1^ protein.

### Statistical analysis

All experiments were carried out using two experimental replicates, and their mean value was considered as one biological replicate. Four biological replicates were used for all samples. Analysis of variance (ANOVA) for quantitative GUS assay data was performed at a probability level of 5% (P≤0.05).

## Results

### Cloning of *SbGSTU*-P and *in-silico* analysis

**Fig 1:**
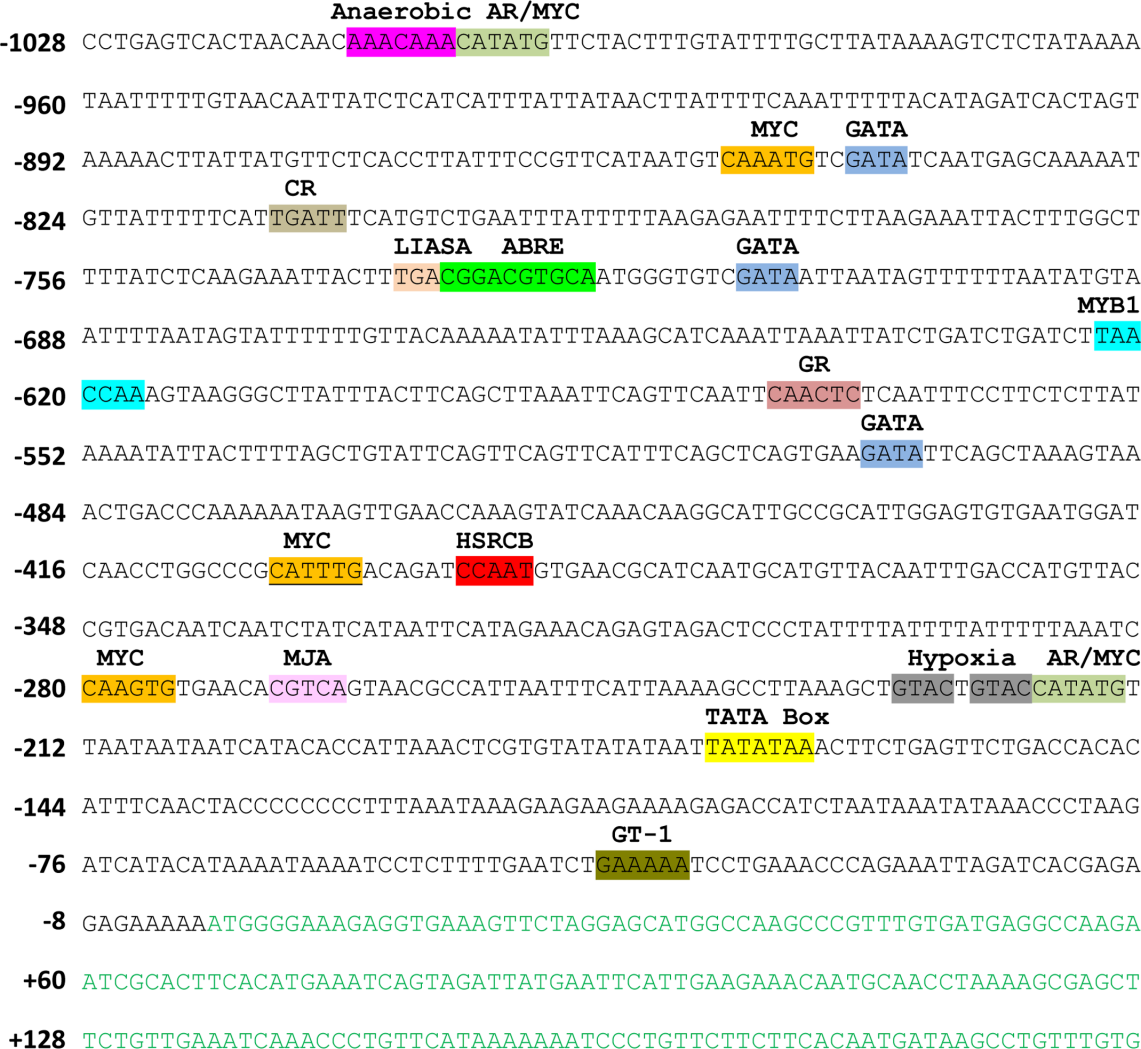
Pictorial representation of *cis*-regulatory motifs on *SbGSTU* promoter. Sequences in green color are the CDS, position of cis-motifs on promoter sequence are represented with colorful highlight and positioned upstream to translation start site. AR: Auxin responsive; CR: cytokinin responsive; LIASA: Light Induced Auxin and Salicylic acid responsive motif; GR: Gibberellin responsive; HSRCB: Heat Shock Responsive CAAT Box. Remaining abbreviation used here are the canonical *cis*-element names.

The putative *SbGSTU* promoter region of 1023 bp (-1028 to -6 bp) was cloned and sequenced (Fig 1). Putative promoter sequence was subjected to homology based *in*-*silico* analysis through online programs PLACE and PlantCARE for the identification of the *cis*-regulatory elements present on the sequences (Fig 1). The nearest predicted TATA box on the promoter sequence starts from 172^nd^ base pair upstream to the ATG codon (Table 1). There were several sites showed homology with the experimentally verified *cis*-regulatory motifs. These motifs were categorized (Table 1) and analysis revealed the presence of at least eight different classes of regulatory motifs, conserved motifs like TATA box and CAAT box, which are essential for the transcription initiation and enhanced level of transcription. Out of different predicted TATA boxes on the promoter region, the motif identified at 172^nd^ base pair upstream of the ATG was the nearest and may be essential for the transcription initiation. Additionally, three Inr motifs responsible for transcription initiation independent of the TATA box were also identified on the plus strand of putative promoter sequences (Table 1). Presence of this motif suggests the diverse mechanism of *SbGSTU* transcription initiation. Other categories of motifs include ABA, dehydration, salinity, light, phytohormones, pathogen/elicitor responsive motifs as well as motifs responsible for developmental stage and organ specific expressions (Table 1). There were six ABA responsive motifs identified of which, two were ABRERELATED1 motif (one on given/shown DNA sequence and one on complementary sequence), one ABRE (on given/shown DNA sequence), two DPBFCOREDCDC3 (both on complementary sequence) and one RYREPEATBNNAPA motif on the complementary sequence. The ABRERELATED1 motif is responsive for ABA as well as dark induced expression for the gene, whereas ABRE provides a direct binding site of AREB transcription factor to initiate expression of the transcript in response to ABA mediated signaling pathway (Table 1). The DPBFCOREDCDC3 motif regulates the transcript expression by binding of the bZIP transcription factor, indirectly regulated by ABA-dependent signaling pathways as similar to the MYB/MYC transcription factors. There was only one MYBAT1 binding site identified whereas 10 MYCCONSENSUSAT were recognized on the putative promoter sequences (Table 1). Two GT1GMSCAM4 motifs, which is known to regulate transcript expression specifically in salinity stress and on pathogen attack in Soybean was also present on the promoter sequence (Table 1). The putative promoter sequence contains light responsive motifs like GATA box, I box and GT-1 consensus, cytokinin, gibberellin, auxin, methyl jasmonate, elicitor and several other pathogen responsive motifs (Table 1).

**Table 1:** Different *cis*-regulatory motifs identified on *SbGSTU* promoter.

Category	Motif	Consensus Sequence	Function	Position
ABA, Dehydration & salinity stress responsive	ABRELATERD1	ACGTG	ABRE-like sequence; ABA and dark-induced senescence	-730 (+), -270 (-)
	ACGTATERD1	ACGT	Etiolation induced expression of erd1	-730 (+), -269 (+), -730 (-), -269 (-)
	ANAERO1CONSENSUS	AAACAAA	One of the 16 anaerobic stress responsive motif	-1011 (+)
	CBFHV	RYCGAC	Cold responsive DRE (Binding site of HvCBF1)	-846 (-), -717 (-)
	CCAATBOX1	CCAAT	CAAT box found in the promoter elements of heat shock proteins	-392 (+), -434 (-)
	CURECORECR	GTAC	Oxygen deficiency responsive gene expression through copper-sensing signal transduction pathway	-228 (+), -223 (+), -228 (-), -223 (-)
	DPBFCOREDCDC3	ACACNNG	ABA inducible bZIP transcription factor DPBF-1 & 2 binding site	-280 (-), -188 (-)
	GT1GMSCAM4	GAAAAA	Salt and pathogen inducible GT-1 motif found in soybean	-6 (+), -43 (+), -820 (-)
	LECPLEACS2	TAAAATAT	Core element of tomato Cys protease binding cis-element	-553 (+)
	MYB1AT	WAACCA	MYB recognition site found in the promoter of dehydration responsive gene rd22	-623 (+)
	MYCCONSENSUSAT	CANNTG	MYC recognition site found in promoter of dehydration responsive genes	-1004 (+), -851 (+), -404 (+), -280 (+), -219 (+), -1004 (-), -851 (-), -404 (-), -280 (-), -219 (-)
	RYREPEATBNNAPA	CATGCA	RY repeat of ABA inducible RY/G box required for seed specific expression	-374 (-)
Conserved promoter motifs	CAATBOX1	CAAT	CAAT promoter consensus sequence	-948 (+), -838 (+), -725 (+), -581 (+), -569 (+), -391 (+), -377 (+), -365 (+), -343 (+), -339 (+), -814 (-), -442 (-), 434 (-)
	INRNTPSADB	YTCANTYY	Inr motif responsible for TATA independent initiation of transcription	-571 (+), -588 (+), -583 (+), -531 (+), -526 (+),-521 (+)
	TATABOX3	TATTAAT	TATA box from sweet potato sporamin A gene	-710 (-)
	TATABOX4	TATATAA	TATA box found in the 5'upstream region of sweet potato sporamin A gene	-180 (+), -172 (+), -173 (-)
	TATABOX5	TTATTT	TATA box found in the 5'upstream region of pea (*Pisum sativum*) glutamine synthetase gene	-923 (+), -870 (+), -823 (+), -795 (+), -607 (+), -298 (+), -293 (+), -963 (-), -473 (-), -123 (-), -66 (-)
	TATABOXOSPAL	TATTTAA	Binding site for OsTBP2	-660 (+), -125 (-)
	TATAPVTRNALEU	TTTATATA	TATA-like motif	-172 (-)
Light responsive	ASF1MOTIFCAMV	TGACG	Light induced auxin and salicylic acid regulated as-1 motif	-736 (+), -268 (-)
	EBOXBNNAPA	CANNTG	E-Box drive light responsive expression	-1004 (+), -851 (+), -404 (+), -280 (+), -219 (+), -1004 (-), -851 (-), -404 (-), -280 (-), -219 (-)
	GATA BOX	GATA	Light responsive expression (found in promoter of all LHCII type I Cab genes	-843 (+), -714 (+), -502 (+), -944 (-), -841 (-), -754 (-), -638 (-), -455 (-), -334 (-)
	GT1CONSENSUS	GRWAAW	GT-1 motif; Light regulated expression	-714 (+), -43 (+), -6 (+), -946 (-), -921 (-), -868 (-), -781 (-), -640 (-), -567 (-), -820 (-), -756 (-)
	IBOXCORE	GATAA	I-box conserved sequence upstream to the light regulated genes	-714 (+), -945 (-), -755 (-), -639 (-)
	SORLIP2AT	GGGCC	Sequences Over-Represented in Light-Induced Promoters	-410 (-)
	SORLREP3AT	TGTATATAT	Sequences Over-Represented in Light-Repressed Promoters	-184 (+)
Phytohormone responsive	ARR1AT	NGATT	Cytokinin regulated ARR1 binding site	-812 (+), -39 (-), -48 (-), -60 (-), -205 (-), -342 (-), -338 (-), -284 (-)
	CAREOSREP1	CAACTC	Gibberellin regulated proteinase expression	-576 (+)
	CATATGGMSAUR	CATATG	Auxin responsive	-1004 (+), -219 (+), -30 (-), -219 (-), -1004 (-)
	CGTCA-motif	CGTCA	Methyl-jasmonate responsive motif	-268 (+), -736 (-)
	CPBCSPOR	TATTAG	Cytokinin regulated protein binding element	-97 (-)
	GAREAT	TAACAAR	GARE (GA-responsive element)	-673 (-)
	MYBGAHV	TAACAAA	GA-inducible MYB protein binding site	-673 (-)
Pollen and embryos specific	-300ELEMENT	TGHAAARK	Endosperm specific	-44 (+), -821 (-)
	AACACOREOSGLUB1	AACAAAC	Endosperm specific	-1014 (+)
	POLLEN1LELAT52	AGAAA	Pollen specific activation	-772 (+), -747 (+), -321 (+), -111 (+), -26 (+), -7 (+), -779 (-)
	GTGANTG10	GTGA	GTGA motif in late pollen gene g10 promoter	-507 (+), -426 (+), -387 (+),-347 (+), -275 (+), -16 (-), -875 (-), -900 (-), -1022 (-)
	GCN4OSGLUB1	TGAGTCA	GCN4 motif required for endosperm specific expression	-1026(+)
Miscellaneous	CARGCW8GAT	CWWWWWWWWG	Binding site of MADS domain protein AGL15	-981 (+), -981 (-)
	DOFCOREZM	AAAG	Core sequence of DOF transcription factor binding site	-975 (+), -655 (+), -618 (+), -491 (+), -459 (+), -242 (+), -234 (+), -119 (+), -108 (+), -54 (-), -127 (-), -543 (-), -739 (-), -758 (-), -764 (-), -993 (-)
	RAV1AAT	CAACA	RAV1 transcription factor binding site	-1015 (+)
	SURECOREATSULTR11	GAGAC	Core of sulphur responsive element	-105 (+), -972 (-)
	UP2ATMSD	AAACCCTA	Up2 motif regulates gene expression during axillary bud outgrowth	-86 (+)
Tissue/organelles specific expression	BOXIINTPATPB	ATAGAA	Box II motifs on some non-consensus type plastid promoters	-323 (+)
	CACTFTPPCA1	YACT	Mesophyll specific expression in C4 plants	-1021 (+),-995 (+),-899 (+), -766 (+), -741 (+), -602 (+), -545 (+), -227 (+), -264 (-), -278(-), -313 (-), -429 (-),-457 (-), -489 (-), -508 (-), -616 (-), -680 (-), -895 (-)
	NODCON2GM	CTCTT	Nodule specific expression	-559 (+), -56 (+), -107 (-), -787 (-)
	OSE2ROOTNODULE	CTCTT	Nodule and organ specific expression after infection	-559 (+), -56 (+), -107 (-), -787 (-)
	RHERPATEXPA7	KCACGW	Root hair specific expression	-16 (+), -730 (-)
	ROOTMOTIFTAPOX1	ATATT	Root specific expression	-661 (+), -549 (+), -501 (+), -696 (-), -662 (-), -550 (-), -91 (-)
	RYREPEATGMGY2	CATGCAT	RY repeat motif	-375 (-)
	RYREPEATLEGUMINBOX	CATGCAY	RY motif (legumin box) of seed storage protein	-375 (-)
	S1FBOXSORPS1L21	ATGGTA	S1F box; repressor of plastid ribosomal protein S1 and L21	-222 (-)
	TAAAGSTKST1	TAAAG	Guard cell specific expression mediated by Dof1 protein	-656 (+), -492 (+), -235 (+), -120 (+), -127 (-)
Pathogen, elicitor and wound responsive	AGMOTIFNTMYB2	AGATCCAA	Wound inducible motif	-396 (+)
	BIHD1OS	TGTCA	Bell like homeodomain transcription factor in disease responses	-854 (+), -346 (-), -400 (-)
	ELRECOREPCRP1	TTGACC	Elicitor responsive element	-361 (+)
	WBBOXPCWRKY1	TTTGACY	W box	-362 (+)
	WBOXATNPR1	TTGAC	WB Box	-737 (+), -401 (+), -361 (+), -853 (-)
	WBOXNTCHN48	CTGACY	W-box found in the Chitinase I and II gene for elicitor responsive expression	-483 (+), -154 (+)
	WBOXNTERF3	TGACY	W box" found in the promoter region of a transcriptional repressor ERF3 gene in tobacco; May be involved in activation of ERF3 gene by wounding	-482 (+), -360 (+), -153 (+), -1024 (-)
	WRKY71OS	TGAC	A core of TGAC-containing W-box; Binding site of rice WRKY71, a transcriptional repressor of the gibberellin signalling pathway; Parsley WRKY proteins bind specifically to TGAC-containing W box elements within the Pathogenesis-Related Class10 (PR-10) genes	-736 (+), -482 (+), -400 (+), -360 (+), -346 (+), -153 (+), -1023 (-), -853 (-), -267 (-)

negative numeral indicate that motifs are upstream of the genes, (+) sign within bracket indicate the location of motif on the presented promoter sequence, (-) sign within bracket after numerals in position column denotes the position of motif on the complementary strand of presented promoter sequence

### Promoter deletion constructs and generation of transgenic tobacco lines

**Fig 2:**
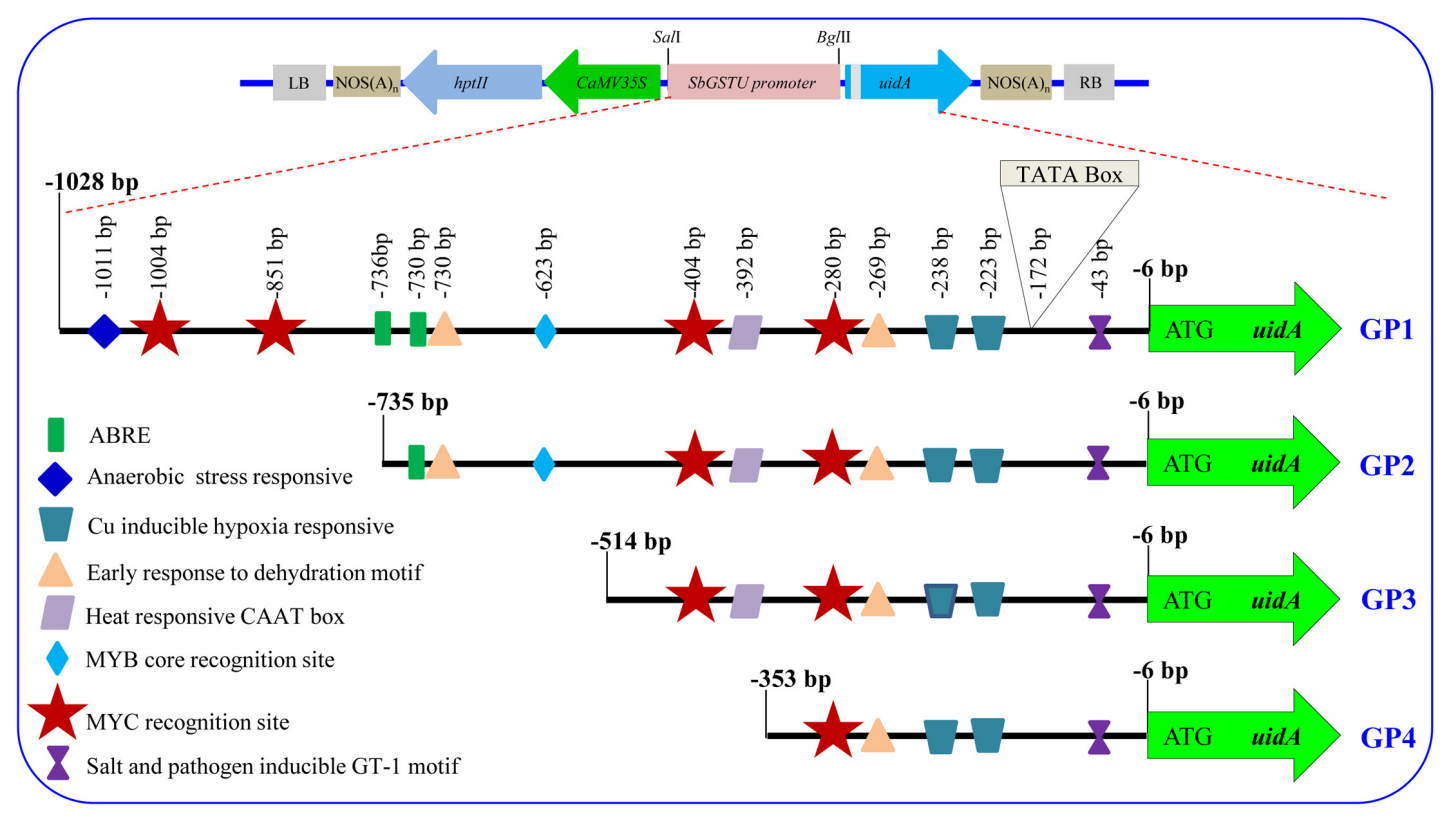
Schematic representation of plant expression vector constructs. The CaMV35S promoter, upstream to *gus* gene, was replaced with different 5’-deletion fragments of *SbGSTU* promoter.

**Fig 3:**
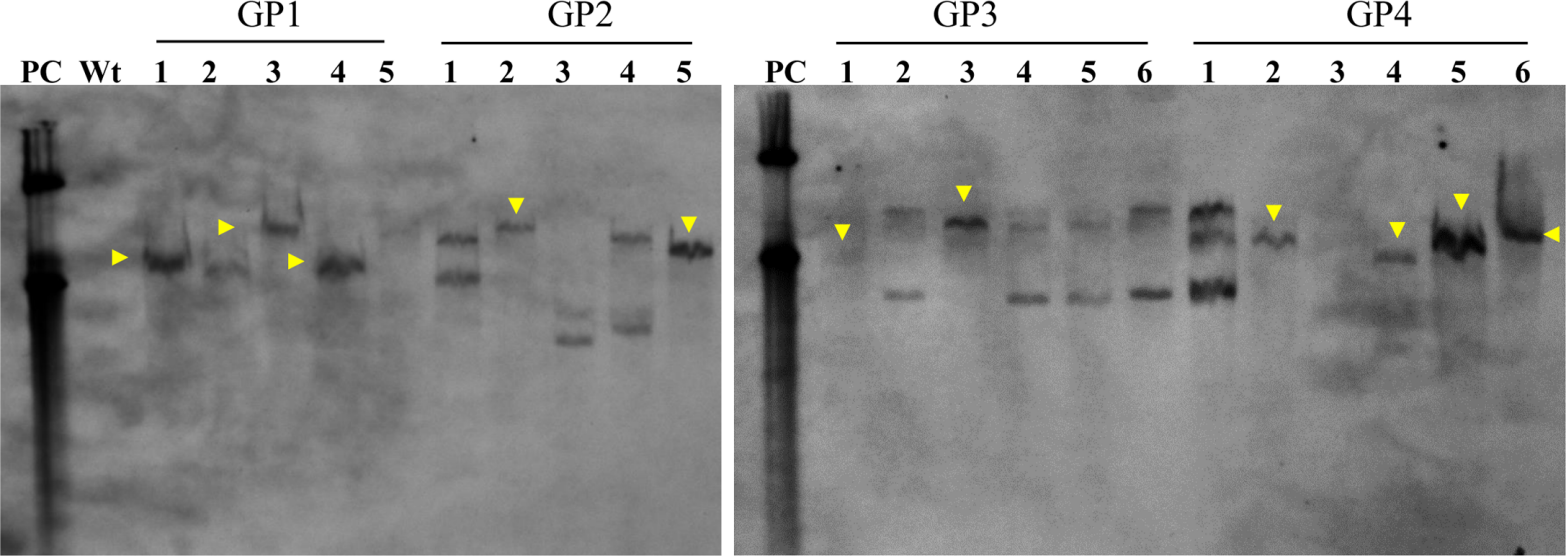
Southern blot analysis of transgenic tobacco lines. Five to six independent transgenic lines of each transformation (GP1-GP4) were randomly selected for transgene event determination.

The putative full-length (-1028 bp to -6 bp upstream of start codon ATG) promoter region including 5’-UTR of the *SbGSTU* was isolated and cloned (GP1; 1023 bp). Three different 5’-deletion constructs of 730 (GP2), 509 (GP3) and 348 bp (GP4) were cloned and sequenced (S1 Fig). Individually, all of these fragments were then sub-cloned into pCAMBIA1301, upstream to *uidA* gene (by replacing CaMV35S promoter; Fig 2) and mobilized into *Agrobacterium tumefaciens* LBA4404 for the transformation of tobacco leaf discs. The transformed leaf discs were regenerated on the selection media and presence of the transgene was confirmed by PCR amplification of the *hptII* gene (S2 Fig). Transgenic tobacco plants were grown under greenhouse conditions and seeds harvested to be used for analysis in the T_1_ generation. Transgenic lines (T_1_) transformed with promoter constructs (GP1–GP4) were analyzed for transgene insertion using Southern hybridization (Fig 3). Total 11 (3, 2, 2 and 4 plants for GP1, GP2, GP3 and GP4, respectively) transgenic lines were found to be single transgene events, whereas other lines have multiple copies. Two plants of each transgenic lines (T_1_) showing single transgene event were selected for further analysis.

### Histochemical GUS assay

**Fig 4:**
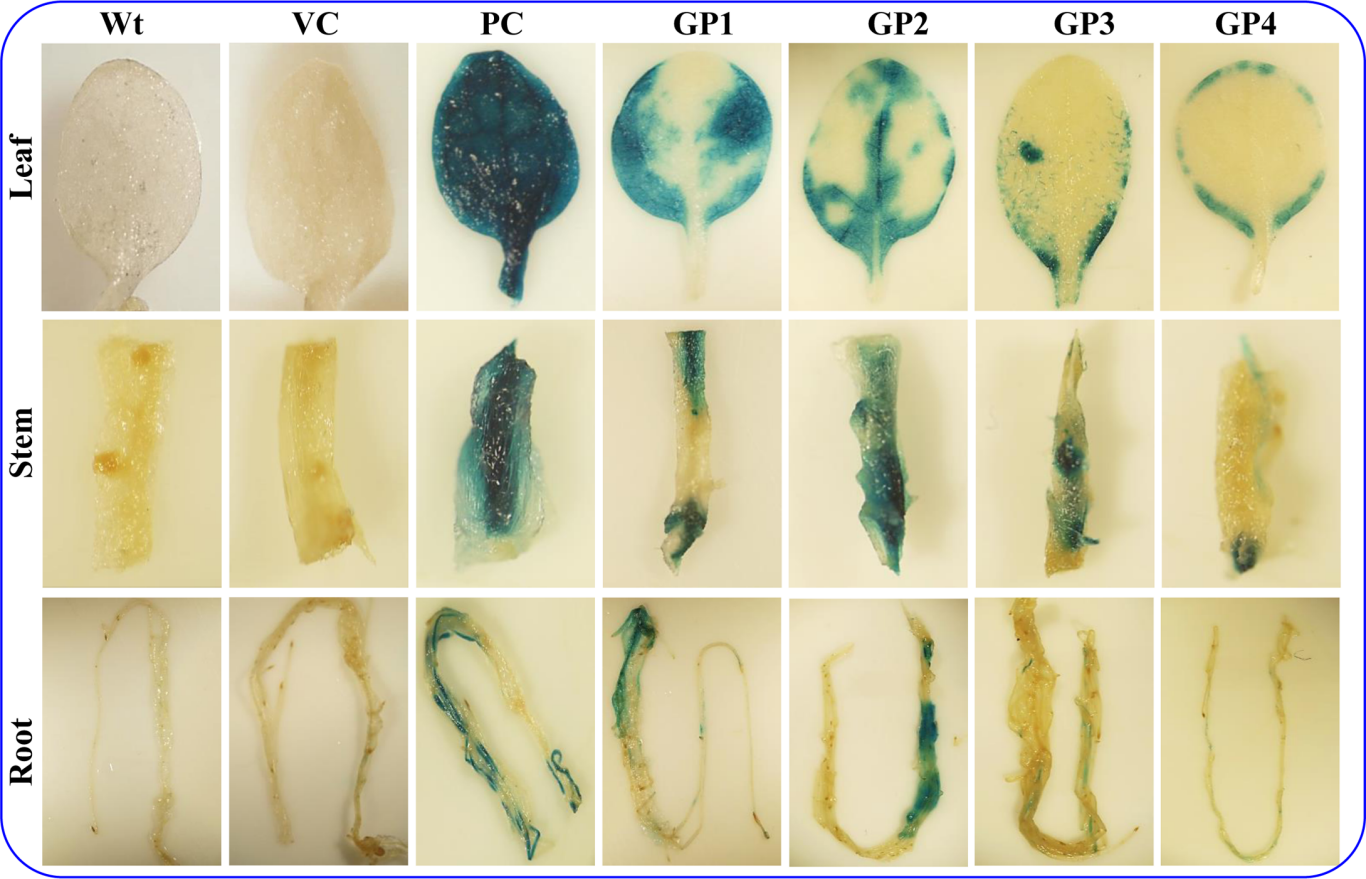
Histochemical GUS assay of T_1_ transgenic tobacco and wild type plants. Transgenic plants were developed by transforming the promoter constructs (GP1–GP4), pCAMBIA1301 (PC), vector control (VC), whereas Wt is wild-type (no transformed) plant.

**Fig 5:**
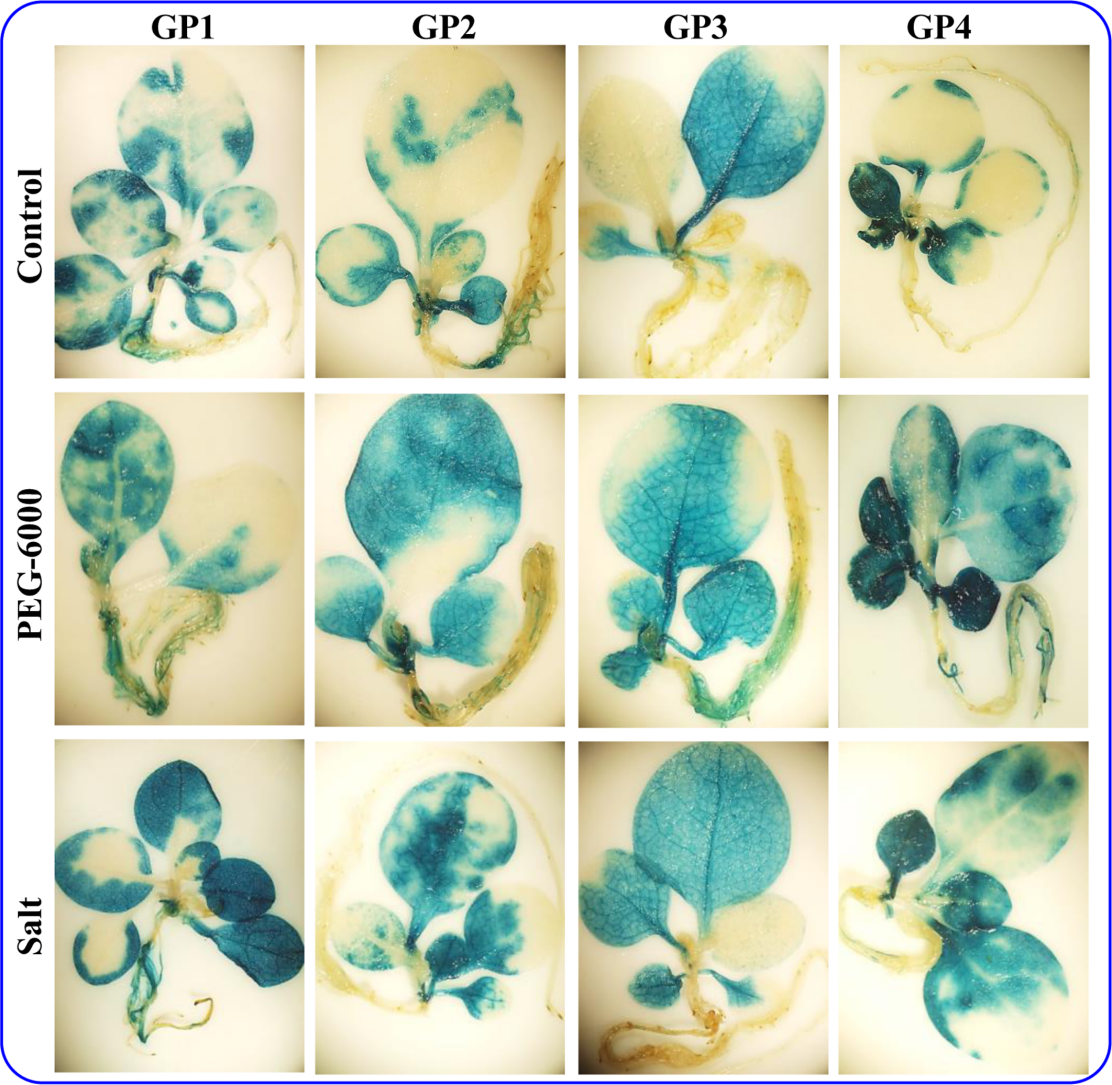
Efficacy analysis of promoter constructs under abiotic stress. Histochemical GUS assay of transgenic plants, developed by transforming the promoter constructs (GP1–GP4) and subjected for salt and osmotic stress was performed and compared with a control condition.

Transgenic plants harboring single copy transgene insertion (of promoter constructs; S3 Fig) were germinated on the hygromycin containing media and after three week growth, were transferred to the hydroponic culture. Expression of the reporter gene driven by putative *SbGSTU* promoter fragments in different plant organs were screened by histochemical GUS assay of the seedling grown in hydroponics for two weeks (Fig 4). There were no expression of the reporter gene in the Wt (wild type, untransformed tobacco plant) and VC, a negative vector control prepared by replacing the CaMV35S promoter with 129 bp of junk vector sequence [34] plants, whereas strong constitutive expression of the GUS protein was observed in all organs, leaf, stem and root in PC plants (Fig 4). The expression level of the GUS in histochemical analysis gradually decreases from GP1 to GP4 in leaf tissues. In the case of stem and root, the highest level of expression was detected in plants transformed with the GP2 construct (Fig 2). To analyze the stress dependent regulation of the promoter fragments, seedlings were subjected to salt (200 mM NaCl) and osmotic (10% PEG-6000) stress. Transgenic plants transformed with the promoter deletion constructs showed a higher level of GUS expression under stress conditions (Fig 5), suggesting its role in abiotic stress by driving enhanced expression of the gene.

### Quantitative MUG assays under salt stress

**Fig 6:**
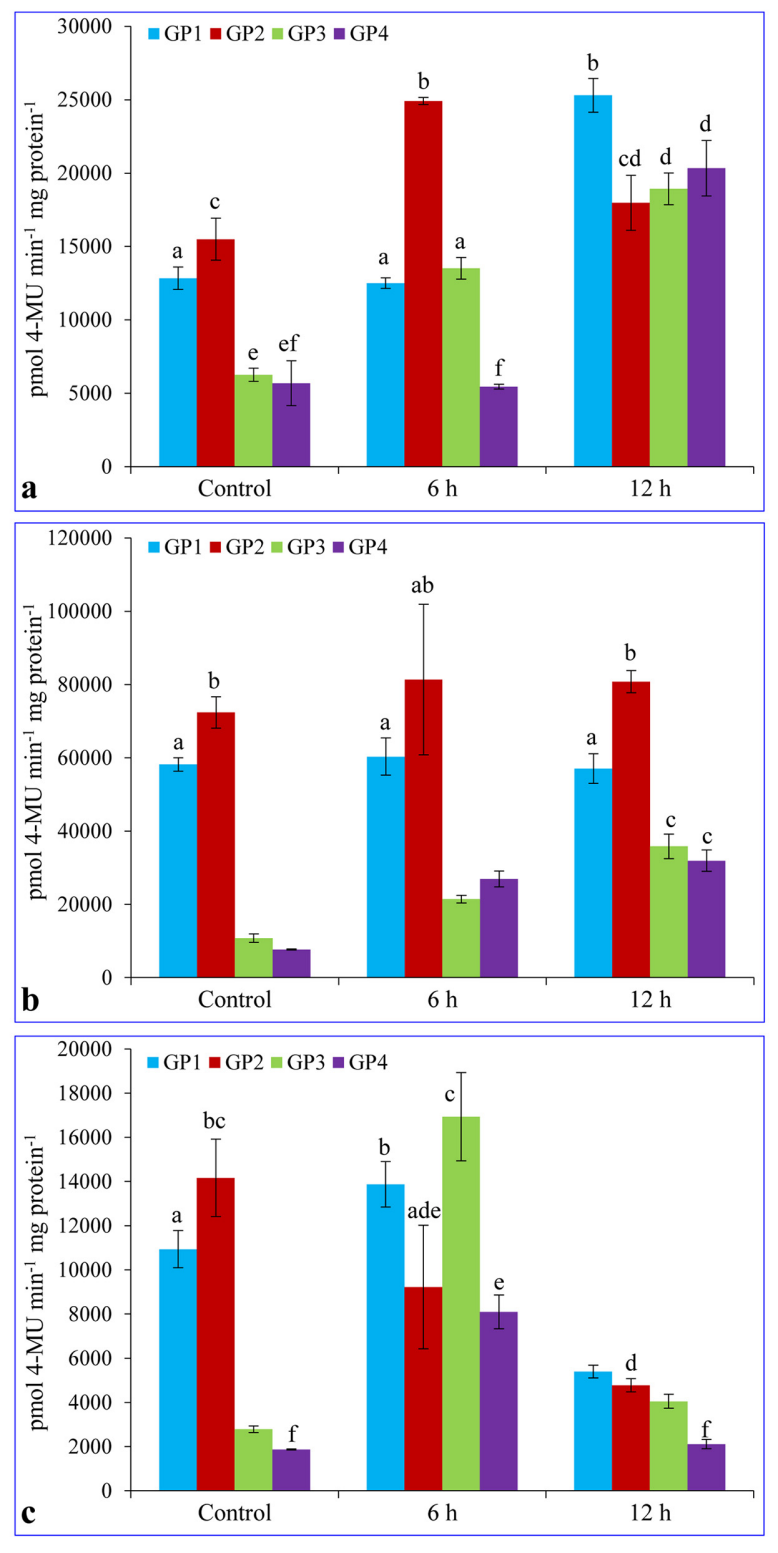
Expression of the *GUS* gene driven by the promoter constructs under salt stress in T_1_ transgenic plants. Five week old transgenic plants (T_1_) were treated with 200 mM NaCl, crude protein from leaves (a), stem (b) and root (c) were extracted and quantification was performed using 4-methylumbelliferyl-beta-D-glucuronide as substrate. Bars represent mean value of GUS enzyme activity ± SD and the bars which share similar letters are non-significant at *p*≤0.5

A fluorescent MUG assay was performed for quantifying the GUS protein expressed by cloned *cis*-regulatory elements of the putative promoters and compared with the control condition (Fig 6 and S2 Table). No significant change was observed in GUS expression level at 6 h in leaf tissues transformed with GP1 construct, but increased approximately 2-fold at 12 h under salinity stress compared to control. The GUS expression level was highest in GP2 transgenic lines under control condition, which increased by 1.6 fold at 6 h of salinity stress treatment and again decreased upto control at 12 h. Plants transformed with GP3 or GP4 constructs showed significant increase of GUS expression at 12 h of treatment, but there were no significant difference in GP2, GP3 and GP4 at this time point (Fig 6A). In stem tissue, plants transformed with the GP1 and GP2 maintained the high but similar level of GUS expression as of control plants. Under control, the expression level in GP3 and GP4 were very low (5-10-fold) compared to GP1 and GP2, but both of them showed stress inducibility and expression level increased concomitantly with increasing time point of stress treatment (Fig 6B). Expression of GUS in root tissues of transformed plants, except GP2, increased significantly at 6 h of salt treatment, thereafter decreased at 12 h time point of stress treatment. In contrast, GUS expression was highest in control plants and reduced concomitantly with each time point in plants transformed with GP2 (Fig 6C).

### Quantitative MUG assays under osmotic stress

**Fig 7:**
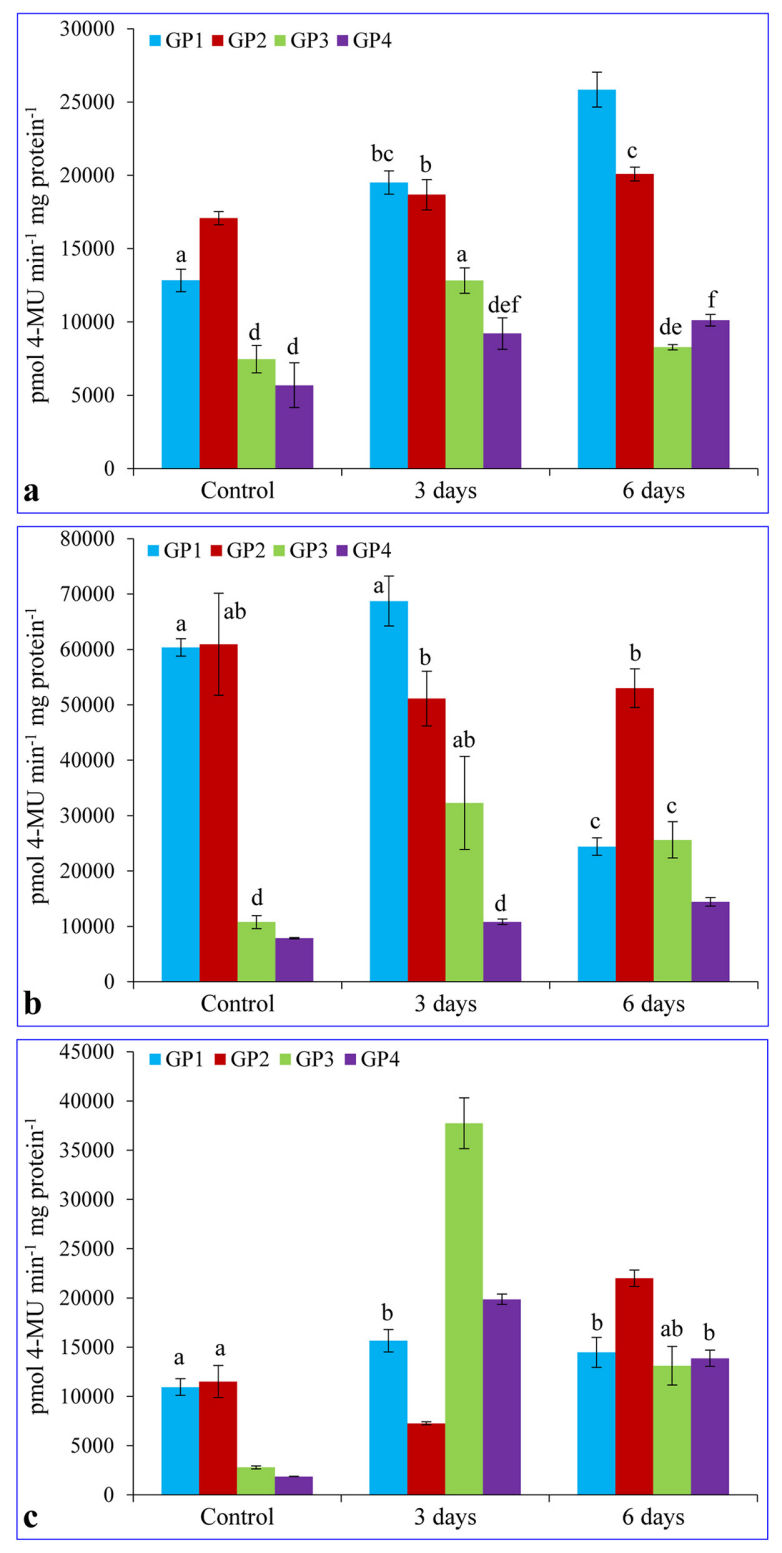
Expression of the *GUS* gene driven by the promoter constructs under osmotic stress in T_1_ transgenic plants. Five week old transgenic plants (T_1_) were treated with 10% PEG, crude protein from leaves (a), stem (b) and root (c) were extracted and quantification was performed using 4-methylumbelliferyl-beta-D-glucuronide as substrate. Bars represent mean value of GUS enzyme activity ± SD and the bars which share similar letters are non-significant at *p*≤0.5

Quantitative MUG study was also performed under osmotic stress with transgenic plants containing transgene controlled by different promoter deletion constructs (Fig 7 and S2 Table). Transgenic plants transformed with GP1 and GP2 constructs and treated with osmotic stress showed a concomitant increase in the GUS expression level with increasing time points in leaves. The level of expression which was found lower in GP1 than GP2 under control exceeded the level in GP2 at 6 days of stress treatments. Although, the GUS expression in GP1 to GP4 increased under osmotic stress, however, a very slight increase was observed in the GP3 and GP4 plants as compared to the GP1 and GP2 (Fig 7A). In stems, a slight increase in the GUS level was observed in the GP3 and GP4, but it was decreased in case of transgenic plants transformed with the GP1 and GP2 (Fig 7B). Quantitative analysis of GUS in root tissues of transgenic plants, subjected to osmotic stress showed increased GUS activity on the 3^rd^ day of stress treatment, but reduced again on the 6^th^ day except in GP2. In GP2, the expression level first decreased on the 3^rd^ day and then reached to its highest level of expression on the 6^th^ day treatment (Fig 7C).

## Discussion

Plants are sessile and cannot escape from the unfavorable environmental conditions. Therefore, they evolved the homeostatic mechanism for their survival in the fluctuating environmental conditions. The occurrence of extreme environmental conditions like high salinity, drought, high temperature and high light intensity are the common stresses and major challenges for the sustainable agriculture. Among different strategies for crop improvement, the transgenic approach has emerged as one of the most rapid for crop engineering with desired character. For genetic engineering of a crop requires a potential source of gene/transgene responsible for desired character and an efficient promoter to drive enough level of expression in the host plants. This study was designed to clone a promoter of stress responsive *SbGSTU* gene from an extreme halophyte *Salicornia brachiata* and its functional validation in tobacco. At the beginning of stress conditions, a lower concentration of generated ROS initiate the defense signaling pathways in plants but with an increase in time and quantity of stress, ROS accumulation exceeds the homeostatic level. Over-accumulation of ROS in cell organelles oxidizes major biomolecules such as proteins, DNA, lipids and carbohydrates in cytotoxic molecules [45]. Now, at this stage, GST turns on and starts the detoxification of these cytotoxic molecules and their degradation.

The expression of genes encoding GSTs is regulated by a number of environmental stimuli. The transcript expression of *SbGSTU* was upregulated in the salinity, drought, cold and ABA treatment, but there was no significant change in expression level observed under salicylic acid treatment [14]. The highest level of transcript upregulation was found in the plants treated with ABA followed by drought stress. Bio-informatics analysis of the *SbGSTU* promoter sequences showed the presence of six ABA responsive motifs, MYB and MYC transcription factor binding sites (Table 1). These all *cis*-regulatory motifs have the ability to converse ABA signaling pathways under osmotic or drought stress conditions and thus may be responsible for the higher transcript expression in these stresses as reported by Jha et al. [14]. Heterologous transgenic approach to study the expression efficiency of *SbGSTU*-P showed a higher level of GUS reporter protein expression even in untreated transgenic tobacco leaves (Fig 5). This finding is in coherence with the previous reports on the comparative transcriptomic analysis of *A*. *thaliana* and its halophytic relative *T*. *halophila*. Plant *T*. *halophila* exhibited constitutively higher expression level of many stress-related genes than that of in *A*. *thaliana*. Halophytes have this unique ability of better transcription efficiency [18–19] and this may be the reason for the higher expression level of GUS reporter protein even under untreated conditions. When transgenic seedlings were subjected to the salinity or osmotic stress conditions, the level of GUS activity increased in all the transgenic plants (Fig 5). It has already been discussed about the presence of six ABA responsive, salinity responsive GT-1 and drought/osmotic stress responsive MYB and MYC binding motifs in the promoter region (Table 1). It is well known that osmotic stress and the ABA signaling pathway converge at SnRK2 proteins and then finally leads to activation of the AREB to induce ABA responsive gene expression [46]. Thus presence of ABA responsive motifs may be the region for increased expression of GUS under stress treatments. In coherence to our findings, the accumulation of *At*GSTU19 was induced by oxidative stress generated due to the drought conditions [47]. Similarly, two tau class GSTs, *Os*GSTU3 and *Os*GSTU4 accumulation in root tissues of rice were induced upon treatment with polyethylene glycol [48].

In the quantitative GUS expression analysis of transgenic plant tissues grown under normal or stress conditions, the level of expression was higher in the GP2 plant leaves under normal or 6 h salinity treated plants, but highest expression was found in the GP1 at 12 h of salinity treated plants (Fig 6A). This result suggests that there may be the presence of regulatory motifs in the region between -1028 to -736 bp which regulates the transcript expression level and deletion of this region in GP2 increased the GUS expression level. Highest expression in GP1 at 12 h time point suggests that two MYC consensus binding sites, located between -1028 to -736 bp regions (Figs 1 and 2; Table 1), are induced in the later stages of stress exposure. Interestingly, there were no change in GUS expression in the GP1 and GP2 stem tissues (Fig 6B), which suggests that presence of five TAAAGSTKST1 motifs (Table 1), specific for guard cell specific expression may have some interaction with the stress responsive motifs and directs expression only in the leaf tissues. In roots, rapid upregulation was observed at the 6 h of stress treatment and then downregulation at the 12 h (Fig 6C). This may be occurred due to root tissues are first plant part exposed to the stress medium. When plants were subjected to osmotic stress conditions, the GUS expression level in leaves of all transgenic plants increased with the time point of stress exposure (Fig 7A). Opposite to the salinity stress, higher expression level in the GP1 leaves revealed that the MYC consensus binding sequences located in the region between -1028 to -736 bp are osmotic stress specific (Figs 1 and 2; Table 1). Similar to the salinity stress conditions, under osmotic stress the expression level of the reporter protein in stems was changed slightly in GP1 and GP2 and in the case of root tissues, the rate of expression was rapid (Fig 7B and 7C).

## Conclusions

In conclusion, the *SbGSTU* promoter showed the presence of a number of abiotic stress responsive, phytohormones responsive, pathogen and wound responsive *cis*-regulatory motifs. The presence of ABRE and MYB transcription factor binding sites on the promoter, suggests that the expression of the *SbGSTU* is regulated by ABA-mediated signaling pathway, under abiotic stresses. Functional analysis of deletion constructs of the promoter using heterologous transgenic approach showed an adequate level of GUS reporter protein in both under control as well as stress treated samples. Even the smallest promoter fragment of 348 bp has the efficient expression level of the reporter protein. This finding enables *SbGSTU* promoter as a strong candidate to be used as plant derived promoter for crop genetic engineering for better abiotic stress tolerance.

## Supporting Information

S1 Table Primers used in the study and PCR conditions. (DOCX)

S2 Table Quantitative expression assay of the GUS gene driven by the promoter constructs under salt and osmotic stress in T_1_ transgenic plants. (DOCX)

S1 Fig PCR amplification of full length promoter and deletion fragments. (PPTX)

S2 Fig Molecular confirmation of transgenic tobacco lines. PCR amplification of the *hptII* gene using DNA templet extracted from putative transgenic tobacco plants transformed with (a) GP1, (b) GP2, (c) GP3 or (d) GP4 promoter constructs.
(PPTX)

S3 Fig Schematic diagram of the plant expression vector constructs. Vector pCAMBIA1301 is used as positive control (PC), vector control (VC) and different promoter constructs (GP1-GP4) were prepared by replacing the CaMV35S promoter (upstream to *gus* gene) with 129 bp of junk vector sequence and different 5’-deletion fragments of *SbGSTU* promoter.
(PPTX)
