# Supplementary material for: Functional Characterization of the Tau Class Glutathione-S-Transferases Gene (SbGSTU) Promoter of Salicornia brachiata under Salinity and Osmotic Stress
Source: PLoS One. 2016 Feb 17;11(2):e0148494. doi: 10.1371/journal.pone.0148494 (PMC4757536; doi:10.1371/journal.pone.0148494)
Supplement: S1 Table — (DOCX) [file pone.0148494.s001.docx]

**S1 Table. Primers used in the study and PCR conditions**

| **Purpose** | **Primer** | **Orientation** | **Sequence (5’-3’)** | **PCR conditions** |
| --- | --- | --- | --- | --- |
| Full length promoter | GSTP F | Forward | CCTGAGTCACTAACAACAAAC | [95°C-5min] x1; [95°C-15sec, 60°C-30sec, 68°C-2min] x35; [68°C-7min] x1 |
|  | GSTP R | Reverse | CTCTCTCGTGATCTAATTTCTG |  |
| Deletion fragments vector constructs | GP1 F | Forward | gtcgacCACTATAGGGCATACTAGAGG | [95°C-3min] x1; [95°C-30sec, 60°C-30sec, 72°C-60sec] x30; [72°C-7min] x1 |
|  | GP2 F | Forward | gtcgacCCTGAGTCACTAACAACAAAC |  |
|  | GP3 F | Forward | gtcgacGACGGACGTGCAATGGGTG |  |
|  | GP4 F | Forward | gtcgacCAGCTCAGTGAAGATATTCAGC |  |
|  | GP R | Reverse | agatctACCATGGCTCTCTCGTGATCTAATTTCTG |  |
| Confirmation of transgenic lines | hpt F | Forward | TTCTTTGCCCTCGGACGAGTG | [95°C-3min] x1; [95°C-30sec, 60°C-30sec, 72°C-60sec] x30; [72°C-7min] x1 |
|  | hpt R | Reverse | ACAGCGTCTCCGACCTGATG |  |
| Probe preparation for Southern hybridization | GQF | Forward | GTGAAGGGCCAACAGTTCC | [95°C-3min] x1; [95°C-30sec, 60°C-30sec, 72°C-40sec] x30; [72°C-7min] x1 |
|  | GQR | Reverse | GGTAATGCGAGGTACGGTA |  |

gtcgac:*Sal*I and agatct: *Bgl*II restriction site
