# Supplementary material for: Functional Characterization of the Tau Class Glutathione-S-Transferases Gene (SbGSTU) Promoter of Salicornia brachiata under Salinity and Osmotic Stress
Source: PLoS One. 2016 Feb 17;11(2):e0148494. doi: 10.1371/journal.pone.0148494 (PMC4757536; doi:10.1371/journal.pone.0148494)
Supplement: S2 Table — (DOCX) [file pone.0148494.s002.docx]

**S2 Table. Quantitative expression assay of the GUS gene driven by the promoter constructs under salt and osmotic stress in T_1_ transgenic plants**

| **Salt stress** | |  |  |  | **Osmotic stress** | |  |
| --- | --- | --- | --- | --- | --- | --- | --- |
| **Leaf** |  |  |  |  |  |  |  |
| **Promoter Constructs↓** | **Control** | **6 h** | **12 h** |  | **Control** | **3 days** | **6 days** |
| GP1 | 12834.85±767.04 | 12505.13±367.85 | 25300.67±1158.81 |  | 12834.85±767.04 | 19510.65±796.39 | 25845.60±1187.27 |
| GP2 | 15497.44±1429.53 | 24912.23±242.64 | 17970.94±1872.61 |  | 17078.30±449.94 | 18678.59±1039.06 | 20096.78±472.59 |
| GP3 | 6256.22±453.70 | 13512.65±737.48 | 18935.95±1081.41 |  | 7461.89±941.21 | 12823.36±872.66 | 8275.00±179.70 |
| GP4 | 5682.16±1526.32 | 5449.51±163.29 | 20339.12±1893.56 |  | 5682.16±1526.32 | 9206.91±1081.25 | 10109.49±397.50 |
| **Stem** |  |  |  |  |  |  |  |
| GP1 | 58187.53±1844.00 | 60332.14±5100.23 | 57079.46±4036.26 |  | 60355.62±1587.05 | 68734.69±4510.52 | 24398.93±1589.75 |
| GP2 | 72395.87±4268.52 | 81366.03±20579.20 | 80784.22±3003.86 |  | 60939.82±9215.03 | 51121.90±4928.41 | 52991.41±3482.24 |
| GP3 | 10778.38±1179.94 | 21429.08±1061.44 | 35851.78±3363.93 |  | 10778.38±1179.94 | 32283.09±8417.70 | 25600.67±3266.11 |
| GP4 | 7657.39±157.11 | 26949.52±2163.83 | 31929.50±2894.64 |  | 7859.44±115.13 | 10812.45±494.53 | 14424.67±780.21 |
| **Root** |  |  |  |  |  |  |  |
| GP1 | 10935.50±845.26 | 13874.93±1025.55 | 5397.63±286.54 |  | 10935.50±845.26 | 15655.87±1144.36 | 14468.68±1520.52 |
| GP2 | 14167.16±1754.59 | 9222.69±2795.97 | 4775.52±296.00 |  | 11507.64±1627.25 | 7266.08±144.27 | 21996.19±840.37 |
| GP3 | 2786.96±147.77 | 16933.35±1996.08 | 4046.55±316.88 |  | 2786.96±147.77 | 37744.04±2583.16 | 13102.51±1966.57 |
| GP4 | 1867.82±24.32 | 8100.63±761.58 | 2112.11±206.29 |  | 1867.82±24.32 | 19863.20±532.90 | 13870.07±815.30 |

Value represent mean value of GUS enzyme activity (pmol 4-methylumbelliferone min^-1^ mg protein^-1^) ± SD
