# Supplementary material for: Functional Characterization of the Tau Class Glutathione-S-Transferases Gene (SbGSTU) Promoter of Salicornia brachiata under Salinity and Osmotic Stress
Source: PLoS One. 2016 Feb 17;11(2):e0148494. doi: 10.1371/journal.pone.0148494 (PMC4757536; doi:10.1371/journal.pone.0148494)
Supplement: S1 Fig — (PPTX) [file pone.0148494.s003.pptx]

## Slide 1
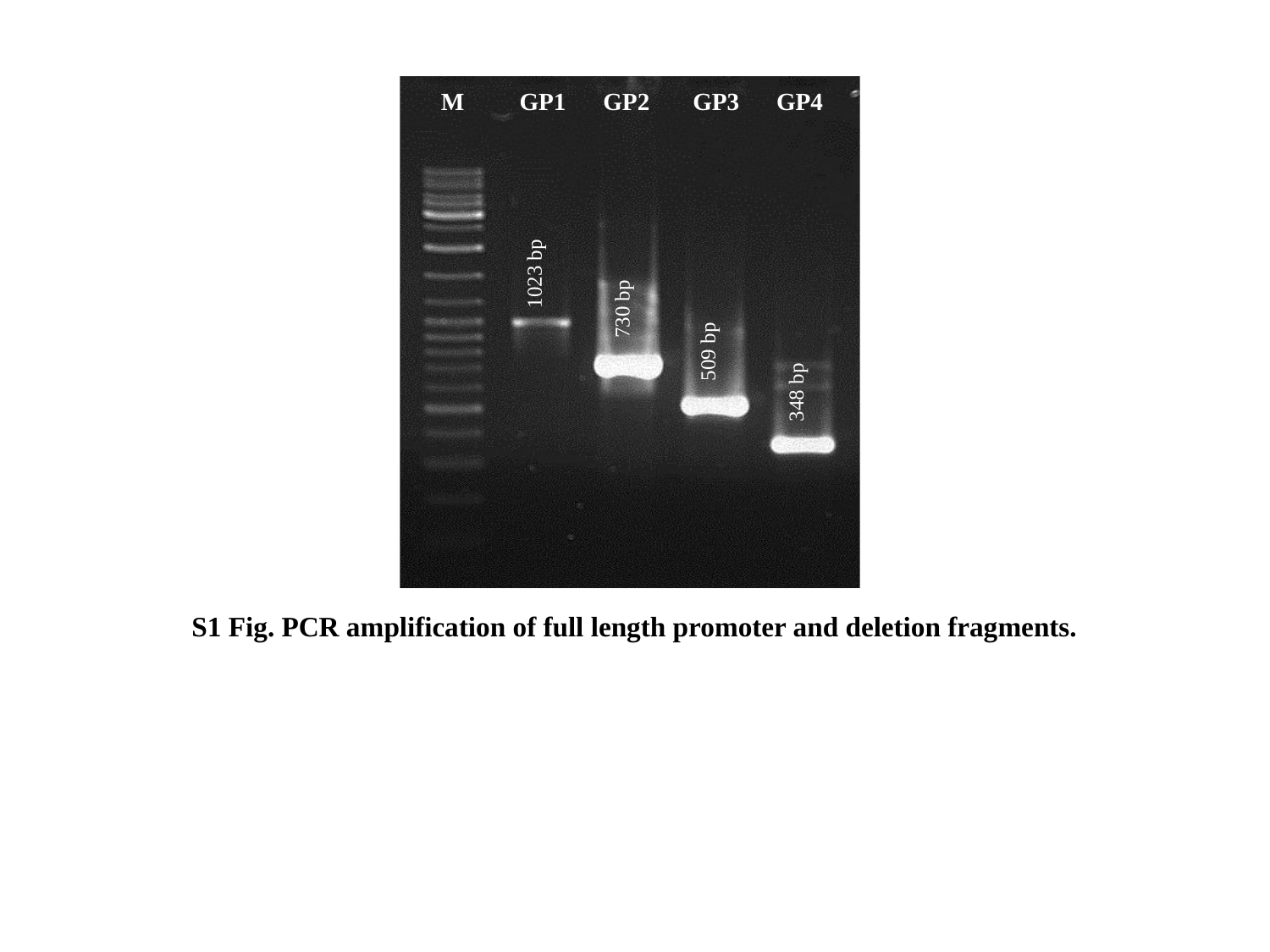

1023 bp
730 bp
509 bp
348 bp
 M GP1 GP2 GP3 GP4
S1 Fig. PCR amplification of full length promoter and deletion fragments.
