# Supplementary material for: Functional Characterization of the Tau Class Glutathione-S-Transferases Gene (SbGSTU) Promoter of Salicornia brachiata under Salinity and Osmotic Stress
Source: PLoS One. 2016 Feb 17;11(2):e0148494. doi: 10.1371/journal.pone.0148494 (PMC4757536; doi:10.1371/journal.pone.0148494)
Supplement: S2 Fig — PCR amplification of the hptII gene using DNA templet extracted from putative transgenic tobacco plants transformed with (a) GP1, (b) GP2, (c) GP3 or (d) GP4 promoter constructs. (PPTX) [file pone.0148494.s004.pptx]

## Slide 1
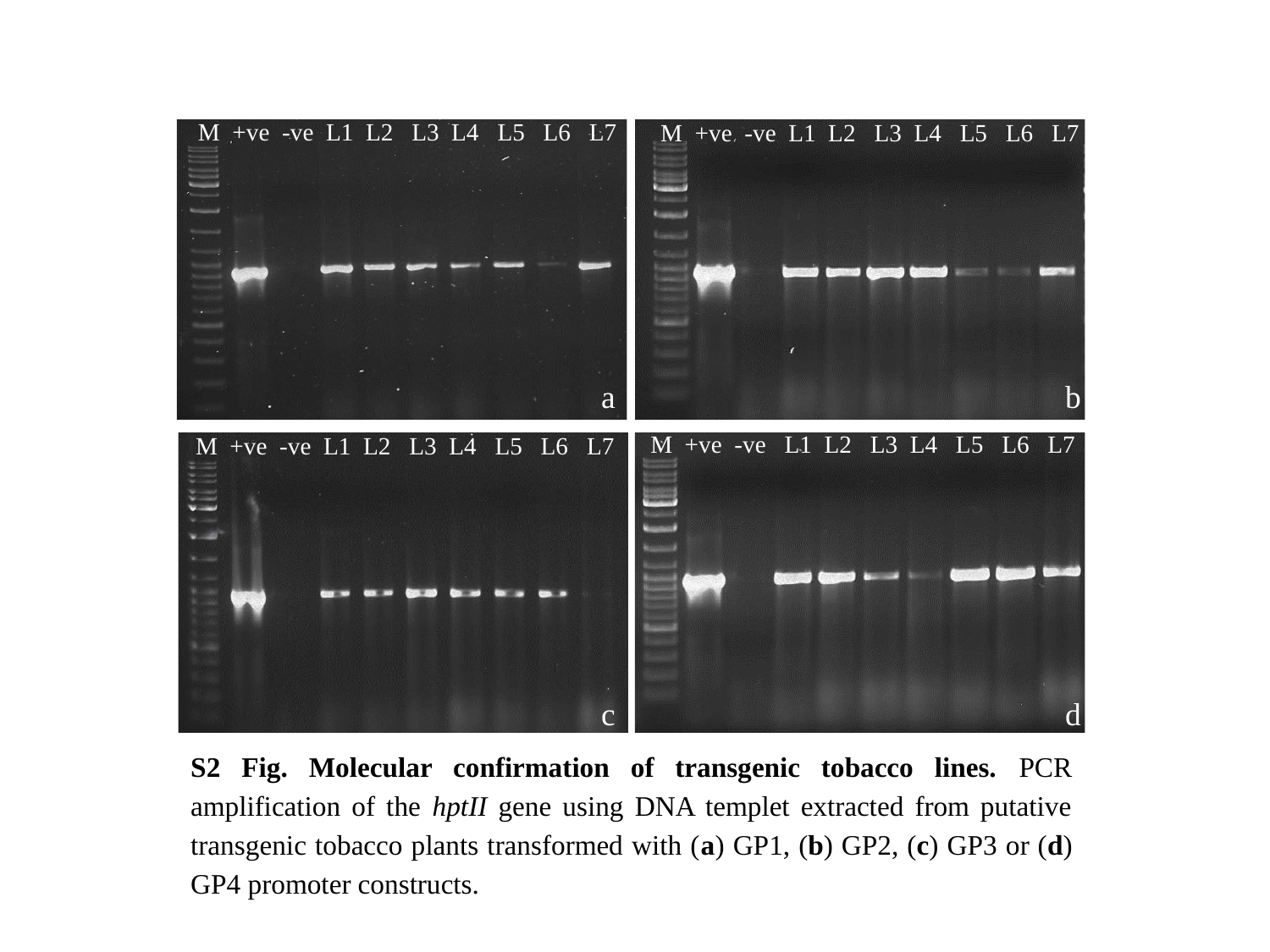

M +ve -ve L1 L2 L3 L4 L5 L6 L7
M +ve -ve L1 L2 L3 L4 L5 L6 L7
M +ve -ve L1 L2 L3 L4 L5 L6 L7
M +ve -ve L1 L2 L3 L4 L5 L6 L7
a b
c d
S2 Fig. Molecular confirmation of transgenic tobacco lines. PCR amplification of the hptII gene using DNA templet extracted from putative transgenic tobacco plants transformed with (a) GP1, (b) GP2, (c) GP3 or (d) GP4 promoter constructs.
