# Supplementary material for: Functional Characterization of the Tau Class Glutathione-S-Transferases Gene (SbGSTU) Promoter of Salicornia brachiata under Salinity and Osmotic Stress
Source: PLoS One. 2016 Feb 17;11(2):e0148494. doi: 10.1371/journal.pone.0148494 (PMC4757536; doi:10.1371/journal.pone.0148494)
Supplement: S3 Fig — Vector pCAMBIA1301 is used as positive control (PC), vector control (VC) and different promoter constructs (GP1-GP4) were prepared by replacing the CaMV35S promoter (upstream to gus gene) with 129 bp of junk vector sequence and different 5’-deletion fragments of SbGSTU promoter. (PPTX) [file pone.0148494.s005.pptx]

## Slide 1
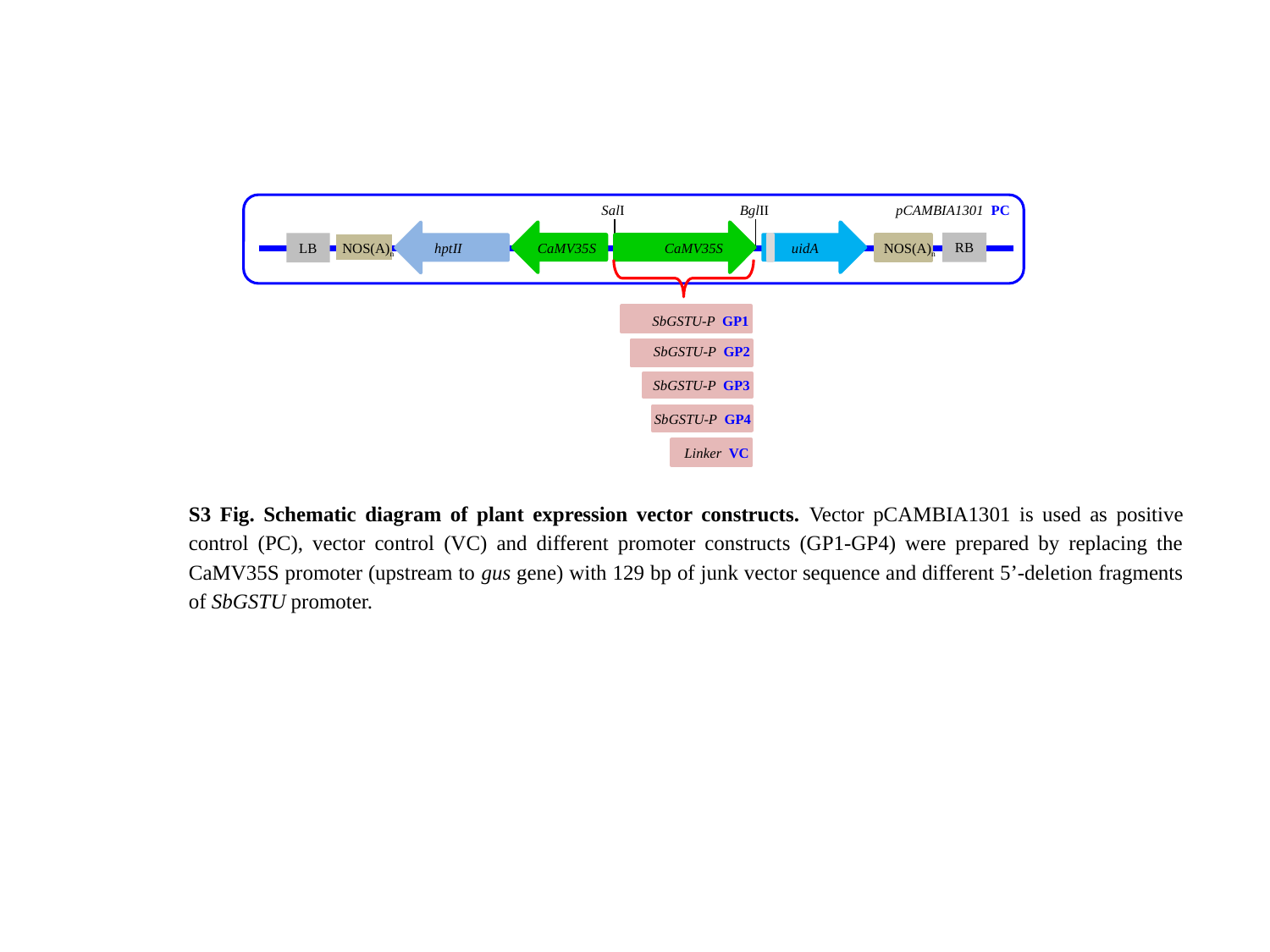

SalI BglII
RB
LB
SbGSTU-P GP3
SbGSTU-P GP4
NOS(A)n hptII CaMV35S CaMV35S uidA NOS(A)n
SbGSTU-P GP1
SbGSTU-P GP2
Linker VC
pCAMBIA1301 PC
S3 Fig. Schematic diagram of plant expression vector constructs. Vector pCAMBIA1301 is used as positive control (PC), vector control (VC) and different promoter constructs (GP1-GP4) were prepared by replacing the CaMV35S promoter (upstream to gus gene) with 129 bp of junk vector sequence and different 5’-deletion fragments of SbGSTU promoter.
